# Exercise enjoyment and school sports: PE, general activity, and sports club participation

**DOI:** 10.3389/fspor.2026.1792875

**Published:** 2026-04-17

**Authors:** Lasse Vogt, Lutz Vogt

**Affiliations:** 1 Sport Sciences, Goethe-Universität Frankfurt, Frankfurt am Main, Germany; 2 Sports Medicine & Exercise Physiology, Goethe-Universität Frankfurt, Frankfurt am Main, Germany

**Keywords:** adolescence, exercise enjoyment, physical activity behavior, physical education, sports club participation

## Abstract

**Background:**

Enjoyment of physical activity is a central determinant of lifelong engagement in exercise and health-oriented behavior.

**Objective:**

The present study examined associations between enjoyment of physical education (PE), general exercise enjoyment, and sports club participation among secondary school students.

**Methods:**

Sixty-two students (18 ♀, 44 ♂; grades 5–10) completed an anonymous survey including the Physical Activity Enjoyment Scale—short version (PACES-S), self-reported PE grades, and current and previous sports club/fitness memberships. Responses on PE enjoyment and perceived performance relative to classmates were collected using four-point scales. Data were analyzed descriptively and inferentially using Jamovi 2.6.26 (*α* = 0.05).

**Results:**

Three-quarters of students reported frequent or consistent enjoyment of PE. This group achieved significantly higher PE grades (1.98 ± 0.89 vs. 2.57 ± 1.16; *p* = 0.045) and higher PACES-S scores (15.5 ± 3.2). Active sports club members reported significantly greater movement enjoyment than non-members (*p* < 0.05). Students who consistently enjoyed PE were five times more likely to report above-average enjoyment of general movement (RR = 5.0; 95% CI: 1.97–12.69) and four times more likely to be active sports club members (RR = 4.36; 95% CI: 1.41–13.42).

**Conclusions:**

Enjoyment of PE is strongly associated with general movement enjoyment and sports club participation. These results underscore the importance of competence-oriented, diverse, and enjoyable PE curricula and suggest that school-sports club collaborations can enhance intrinsic motivation, self-efficacy, and social engagement, promoting sustained physical activity in youth.

## Introduction

Physical education is consistently rated among the most popular school subjects for both boys and girls (1). Current guidelines from the Standing Conference of the Ministers of Education and Cultural Affairs (KMK) and the German Olympic Sports Confederation (DOSB) highlight the pedagogical aim of fostering students’ enjoyment of movement and understanding the health benefits of regular physical activity (2). Consequently, PE is intended to promote intrinsic motivation and sustained engagement in physical activity among children and adolescents.

Early establishment of enjoyment in movement and sports is emphasized as a key factor for lifelong active lifestyles (1). Movement enjoyment can influence both physical domains (e.g., muscle strength, proprioception, range of motion) (3) and psychological domains (e.g., mood, health-related quality of life), thereby impacting behavior, well-being, and holistic development. A commonly used, reliable, and validated instrument to operationalize enjoyment of physical activity is the Physical Activity Enjoyment Scale (PACES) (4, 5). The urgency of examining the links between exercise enjoyment, physical education, and organized sports lies in understanding how positive experiences in both school and club settings promote sustained physical activity. For example, in all-day schools (Ganztagsschule), partnerships with local sports clubs can enhance enjoyable, skill-based activities, supporting long-term engagement (6, 7).

Although school-based PE represents only one of several movement-related contexts for adolescents, systematically assessing students’ subjective enjoyment in both school and general settings, and analyzing its associations with related factors, can provide valuable insights into promoting lifelong physical activity (1).

## Methods

### Participants

Participants included 62 students from grades 5–10 (18 female, 44 male), with the majority (90%) from grades 6–9. Two students were in grade 5 and five in grade 10. The study was conducted in accordance with the Declaration of Helsinki and approved by the local institutional ethics committee (number 2025–59). School administration and parents provided consent for an anonymous survey.

### Measures

The survey collected data on current and past club/fitness membership, last PE grade, and self-assessment regarding PE enjoyment and performance relative to peers. Responses to enjoyment items were measured on a four-point scale (never, sometimes, often, always). General enjoyment of physical activity was assessed using the German short version of the PACES-S (5, 8), a four-item, five-point Likert scale.

### Data analysis

Descriptive and inferential statistics were calculated using MS Excel and Jamovi 2.6.26. Statistical significance was set at *α* = 0.05. Spearman's rank correlation coefficients were used to assess associations between continuous or ordinal variables, including correlations between PACES-S scores, physical education (PE) grades, and sports club participation. For categorical comparisons and risk estimation, point estimates of relative risk (RR) with corresponding 95% confidence intervals were calculated. In this context, relative risks were computed to examine the likelihood of high enjoyment and sports club membership outcomes.

## Results

Sixty-six percent of students were current members of sports clubs, with boys significantly more likely to be members (*p* = 0.034). Eighteen percent were former club members, and 68% reported no active membership in a fitness facility; 16 students reported current fitness club participation. Soccer was the most common extracurricular activity (16%), followed by cycling, show dance, parkour, strength training, and handball (each 5 students).

Three-quarters of students reported often or always enjoying PE, while 13 sometimes and one never enjoyed it. Students who often or always enjoyed PE achieved significantly better PE grades (1.98 ± 0.89) than those with lower enjoyment (2.57 ± 1.16; *p* = 0.045). Boys rated their PE performance relative to peers significantly higher than girls (*p* = 0.039). PE grades predominantly ranged between 1 and 3, with very few grades below 3.

**Figure 1:**
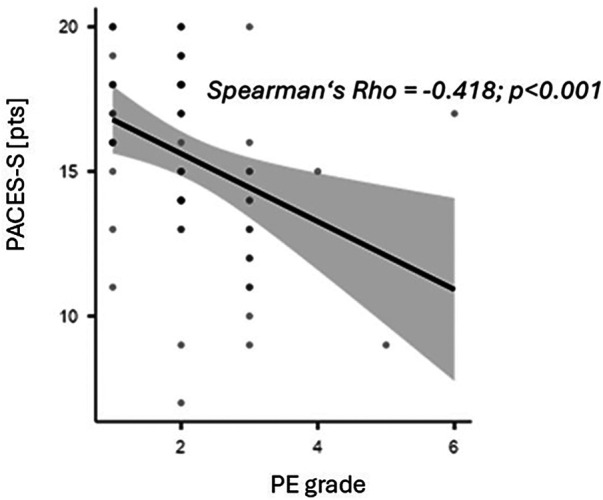
Linear relationship between the most recent school sports grade (PE grade) and overall enjoyment of physical activity (PACES-S).

PACES-S scores averaged 15.5 ± 3.2, with no gender differences observed. A moderate negative correlation between PE grade and PACES-S score was found (*p* < 0.001) (Figure 1).

Active sports club members demonstrated significantly higher PACES-S scores compared to former (*p* = 0.012) or non-members (*p* = 0.037). Students consistently enjoying PE had five times higher likelihood of above-average general movement enjoyment (RR = 5.0; 95% CI: 1.97–12.69) and were four times more likely to be active sports club members (RR = 4.36; 95% CI: 1.41–13.42).

## Discussion

The recent study examined the interplay between school PE enjoyment, general movement enjoyment, and organized sports participation among secondary students. Results indicate a clear association between subjective enjoyment in PE, general activity enjoyment, and PE performance, highlighting that students who enjoy movement tend to perform better academically in PE and engage more in organized sports. The present findings further demonstrate a strong linear relationship between school-based and extracurricular physical activity and exercise enjoyment, emphasizing enjoyment as a central pedagogical target in physical education.

The co-occurrence of enjoyment in PE, participation in sports clubs, and general exercise enjoyment underlines the association between structured and unstructured physical activity, suggesting a mutually reinforcing relationship. This supports initiatives promoting collaboration between schools and sports clubs to provide diverse, competence-building, and intrinsically motivating opportunities for physical activity (9). At the same time, the relatively small sample, drawn from a limited school context, may restrict the generalizability of the findings and limit the robustness of the relative risk estimates.

Although boys reported higher relative performance in PE, no gender differences in overall movement enjoyment were observed, suggesting that both boys and girls benefit equally from enjoyable PE experiences. Previous research indicates that fostering physical literacy, self-confidence, and motor competence contributes to sustained participation and positive emotional experiences in PE (10, 11). However, high competence alone does not guarantee engagement without intrinsic interest (12).

To meet current educational and health promotion guidelines (2), PE curricula should not only strengthen motor competence but also develop additional skills such as movement regulation and self-monitoring, thereby supporting self-determined, health-promoting physical activity (13, 14). Fostering autonomy, competence, and social integration seems essential for creating joyful, sustainable, and lifelong health-enhancing physical activity. In secondary schools, partnerships with extracurricular sports programs can further strengthen these curricular efforts by providing additional activity opportunities and creating informal learning environments that reinforce autonomy, competence, and social integration (15, 16). European initiatives like the *Active School Community* approach demonstrate that such networks reduce barriers to physical activity and promote lasting positive effects on behavior and health attitudes (17). Research shows that embedding school-based physical activity promotion within broader school and community contexts is associated with improved adolescent health outcomes, including social well-being and psychosocial development, which are particularly relevant during the secondary school years (18, 19). Narrative reviews highlight that school-based team sports and collaborative programs contribute to holistic student wellness, encompassing physical, social, and emotional dimensions of health, supporting both academic engagement and lifelong health-promoting behaviors in adolescents (20).

Nevertheless, the interpretations of our study's findings should be made with caution. Because our investigation used a cross-sectional design, we cannot determine the causal direction of the observed associations between enjoyment, performance, and participation in physical activity. Future longitudinal studies are needed to replicate these results, complementing self-reported measures with objective assessments and clarifying potential causal pathways between enjoyment, performance, and sustained participation in organized and non-organized physical activity.

## Conclusion

Enhancing enjoyment in PE not only fosters immediate engagement but also increases the likelihood of general exercise enjoyment and organized sports participation, highlighting the strong link between school-based and extracurricular physical activity.PE curricula that emphasize competence, autonomy, and social engagement are particularly warranted, especially to counteract declining activity levels in adolescent girls (20). Competitive formats should be carefully integrated to prioritize skill development, achievement experiences, and positive social interaction, thereby also enhancing the probability of joyful, health-promoting PE experiences and lifelong physical activity. However, the cross-sectional design precludes causal inferences, and future studies using longitudinal, experimental, or mixed-method approaches with objective measures are needed to further explore these relationships.

## Data Availability

The raw data supporting the conclusions of this article will be made available by the authors, without undue reservation.

## References

[1] Neuber N Kehne M. Freude an Bewegung und Sport früh verankern–Perspektiven für die Entwicklung des Kinder- und Jugendsports. In: Forum Kinder-und Jugendsport. Berlin: Springer (2024). p. 156–64.

[2] KMK & DOSB. Handlungsempfehlungen der Kultusministerkonferenz und des Deutschen Olympischen Sportbundes zur Weiterentwicklung des Schulsports 2023–2028 (2023). Available online at: https://www.kmk.org/fileadmin/veroeffentlichungen_beschluesse/2023/2023_11_10-Schulsport_KMK-DOSB_2023-2028.pdf (Accessed Dezember 12, 2025).

[3] Kashikar-Zuck S Thomas S Bonnette S Gibler RC DiCesare C Schille A Comparison of pain characteristics, strength, and movement patterns in adolescents with juvenile fibromyalgia and high versus low fear of movement. J Pain. (2024) 25(9):104586. 10.1016/j.jpain.2024.104586 38823603 PMC11347078

[4] Jekauc D Voelkle M Wagner MO Mewes N Woll A. Reliability, validity, and measurement invariance of the German version of the physical activity enjoyment scale. J Pediatr Psychol. (2013) 38(1):104–15. 10.1093/jpepsy/jss088 22946084

[5] Jekauc D Nigg C Nigg CR Reichert M Krell-Roesch J Oriwol D Measurement properties of the German version of the physical activity enjoyment scale for adults. PLoS One. (2020) 15(11):e0242069. 10.1371/journal.pone.0242069 33206685 PMC7673501

[6] Noetzel I Becker L Gräfin v. Plettenberg E Kehne M. Forschungsstand zu Bewegung, Spiel und Sport im schulischen Ganztag in Deutschland: Ein Scoping Review. In: Forum Kinder-und Jugendsport. Berlin: Springer (2024). p. 70–83.

[7] Joung K Jeon W Kwon G. Relationship between perceived enjoyment, exercise commitment and behavioral intention among adolescents participating in “school sport club”. Front Med. (2024) 10:1277494. 10.3389/fmed.2023.1277494 PMC10797038 38249984

[8] Chen C Weyland S Fritsch J Woll A Niessner C Burchartz A A short version of the physical activity enjoyment scale: development and psychometric properties. Int J Environ Res Public Health. (2021) 18(21):11035. 10.3390/ijerph182111035 34769552 PMC8582913

[9] Hessisches Kultusministerium. Schule und Sportverein – Gemeinsam für einen bewegten Tag (2015). Available online at: https://kultus.hessen.de/sites/kultus.hessen.de/files/2021-08/hkm_schuleverein-endformat.pdf (Accessed Dezember 12, 2025).

[10] Öztürk Ö Aydoğdu O Kutlutürk Yıkılmaz S Feyzioğlu Ö Pişirici P. Physical literacy as a determinant of physical activity level among late adolescents. PLoS One. (2023) 18(4):e0285032. 10.1371/journal.pone.0285032 37115768 PMC10146507

[11] Woolley A Houser N Kriellaars D. Investigating the relationship between emotions and physical literacy in a quality physical education context. Appl Physiol Nutr Metab. (2024) 49(12):1658–65. 10.1139/apnm-2024-0082 39159488

[12] Yan W Chen L Wang L Meng Y Zhang T Li H. Association between enjoyment, physical activity, and physical literacy among college students: a mediation analysis. Front Public Health. (2023) 11:1156160. 10.3389/fpubh.2023.1156160 37397741 PMC10313414

[13] Sudeck G Rosenstiel S Carl J Pfeifer K. Bewegungsbezogene Gesundheitskompetenz–Konzeption und Anwendung in Gesundheitsförderung, Prävention und Rehabilitation. In: Rathmann K Dadaczynski K Okan O Messer M, editors. Gesundheitskompetenz. Berlin: Springer (2023). p. 33–44.

[14] Brehm W Bös K Graf CH Hartmann H Pahmeier I Pfeifer K Sport als Mittel in Prävention, Rehabilitation und Gesundheitsförderung. Berlin: Springer (2013). 10.1007/s00103-013-1798-y 23978982

[15] Van Acker R De Bourdeaudhuij I De Martelaer K Seghers J Kirk D Haerens L A framework for physical activity programs within school–community partnerships. Quest. (2011) 63(3):300–20. 10.1080/00336297.2011.10483683

[16] Peralta LR O’Connor D Cotton WG Bennie A. The effects of a community and school sport-based program on urban indigenous adolescents’ life skills and physical activity levels: the SCP case study. Health (N Y). (2014) 6(18):2469. 10.4236/health.2014.618284

[17] Phillpots L Grix J. New governance and physical education and school sport policy: a case study of school to club links. Phys Educ Sport Pedagogy. (2014) 19(1):76–96. 10.1080/17408989.2012.726981

[18] Viskari T Appelqvist-Schmidlechner K Ståhl T Vaara JP Fröjd S. Association between school-based physical activity promotion and mental health: a population-based cross-sectional study of schoolchildren in Finland. Eur J Public Health. (2026):ckag020. 10.1093/eurpub/ckag020 41678564 PMC13064530

[19] Jago R Salway R House D Beets M Lubans DR Woods C Rethinking children's physical activity interventions at school: a new context-specific approach. Front Public Health. (2023) 11:1149883. 10.3389/fpubh.2023.1149883 37124783 PMC10133698

[20] Kang X Meng Q Su CH. School-based team sports as catalysts for holistic student wellness: a narrative review. Behav Sci. (2024) 14(7):528. 10.3390/bs14070528 39062351 PMC11274076

